# Utilization and determinants of long term and permanent contraceptive methods among married reproductive age women at Janamora district, northwest Ethiopia

**DOI:** 10.1186/s13104-018-3942-0

**Published:** 2018-11-26

**Authors:** Daniel Shitu Getahun, Haileab Fekadu Wolde, Kindie Fentahun Muchie, Hedija Yenus Yeshita

**Affiliations:** 1 Pneumonia Project Janamora District, College of Medicine and Health Sciences, University of Gondar, Gondar, Ethiopia; 2 Department of Epidemiology and Biostatistics, Institute of Public Health, College of Medicine and Health Sciences, University of Gondar, Gondar, Ethiopia; 3 Department of Reproductive Health, Institute of Public Health, College of Medicine and Health Sciences, University of Gondar, Gondar, Ethiopia

**Keywords:** Prevalence, Determinants, Utilization, Long acting and permanent, Contraceptive

## Abstract

**Objective:**

This study is aimed at determining the prevalence and factors associated with utilization of long acting and permanent methods among married reproductive age (15–49) females at Janamora district, in 2018.

**Result:**

Prevalence of long acting and permanent contraceptive method utilization was 12.9% (95% confidence interval (CI) 10%, 15%).Of those utilizers, 96.8% use implants, 2.1% use female sterilization and 1.1% use Intrauterine Contraceptive Device. Women’s occupation, student as compared to housewife (Adjusted odds ratio (AOR) = 3.12, 95% CI 1.05–9.29), a women whose husband was government employed as compared to merchant (AOR = 2.51, 95% CI 1.1–5.75), and women who had high knowledge as compared to poor knowledge (AOR = 4.20, 95% CI 1.32–13.39) were positively associated with utilization of long acting and permanent contraceptive method.

## Introduction

Contraceptive methods used for family planning can be classified into two categories: long-acting and permanent methods (LAPMs) that are used to limit childbearing and, short-term methods that are used to postpone childbearing [1, 2].

Family planning is central to reduce poverty, promote economic growth, raise female productivity, lower fertility and better child survival and maternal health [3].An estimated 550,000 women die every year through unsafely induced abortion, pregnancy and childbirth. At least 35% of these died due to pregnancies that would be avoided if contraceptives were available [4].Covering the unmet need for modern contraceptive in developing countries like Ethiopia could prevent 54 million unintended pregnancies, 16 million unsafe abortions and 7 million miscarriages. In addition this could also prevent 79,000 maternal deaths and 1.1 million infant deaths [5].

Use of family planning in Ethiopia has traditionally focused on short-acting methods such as injectable and birth control pills. According to Ethiopian Demographic and Health Survey (EDHS) 2016 in Amhara region only 0.5% female sterilization, 3% IUCD, 12.1% implants the rest use short acting contraceptives [6]. High discontinuation rates are associated with the short-acting hormonal methods, such as injectable, that are predominantly chosen by contraceptive users in sub-Saharan Africa. However, increasing contraceptive method mix has been shown to reduce discontinuation rates [7].

Some of the benefits of LAPMs include convenience, high efficacy, high effectiveness (> 99%) cost effectiveness, time, effort, and money saving along with other potential health benefits they can give also uninterrupted protection for the women [8].

If LAPMs are used more effectively, unintended births and induced abortions could be substantively reduced to help families and countries achieve their health goals [9] Different studies conducted in Ethiopia showed the prevalence for utilization of LAPMs to be between 7.3% and 42.98 [2, 4, 10–14].

Utilization of LAPMs are affected by different factors like level of education, income, number of alive children, occupation, age of the women, information and knowledge about LAPMs, joint decision on fertility and attitude towards LAPMs [4, 10, 13–15].

Majority of the studies in our setting focus on the determinants of all modern contraceptive methods in general, but this study aimed to measure the prevalence and determinants of LAPMs utilization among married reproductive aged women in at Janamora district. Therefore this study will create a good insight for health care stakeholders for eradication of factors that contribute for poor utilization of LAPMs.

## Main text

### Methods

The study was conducted at Janamora district from January 1–20, 2018. Janamora is found in North Gondar Zone and it is located 202 kilo meters (km) from Gondar town in north east direction. The total population of the district is 208, 719. Of these 103,942 are females and among the females 24,530 are married and of reproductive age group (15–49) [16].

Community based cross-sectional study design was conducted. A sample size of 761 was determined using single population proportion formula with an assumption of 95% CI, 3% margin of error, 10% non-response rate, 1.5 design effect and utilization of long acting and permanent contraceptive 12.3% [17]. Using a multistage sampling, 7 out of 34 Kebles (smallest administrative levels) of Janamora district were selected by lottery method. Then the calculated sample size was proportionally allocated to each selected Keble. Finally, the eligible study participants were selected by systematic random sampling with a skip interval of eight from prepared sample frame of households. When more than one eligible woman was found in selected households, only one of them was selected by lottery method and if no eligible participants exist in selected households, the next households were considered and all the study participants were above the age of 18.

The study populations for this study were all married and of reproductive age group women’s. Those women, who were seriously ill, cannot talk and support themselves and women with known history of infertility were excluded from the study.

Data were collected using structured interviewer administered questionnaire adopted from literatures and contextualized to the local situations based on the study objectives. The data were collected by five nurses with a qualification of diploma and it was supervised by two health officers. To control for the data quality the tool was pre tested on 5% of the actual sample size out of the study area and training was given to the data collectors. The filled formats were checked daily by the supervisors.

The dependent variable for this study was utilization of long acting and permanent contraceptive method which was defined as women using at least one of LAPMs that prevent pregnancy for more than or equals to 3 years per application. LAMPS include implants, IUCD, male and female sterilization [18]. The explanatory variables include socio economic factors (age, religion, ethnicity, discussion with family about contraceptive, monthly income, occupation), reproductive factors (history of pregnancy, number of alive children, desire for more children), source of information about LAPMs and knowledge towards LAPMs. Knowledge towards LAPMs was assessed by using ten item questions. Based on these questions those women who scored 80% and above from the knowledge questions were categorized as having a good knowledge, 60–79% as moderate knowledge and less than 60% as poor knowledge [19]. Attitude towards LAPMs was measured by using nine item questions. Accordingly, those women who scored above the mean for the correct answers was considered as having positive attitude and women who scored less than or equal to the mean was considered as having negative attitude towards LAPMs [19].

Data were entered using EPI-Info version 7 and analyzed using SPSS version 20. Descriptive statistics like frequencies and percentages were computed for the independent variables in relation to the outcome variable. Binary logistic regression model was fitted to show the association of explanatory variables with utilization of LAPMs. Those variables found to have p-value < 0.2 in the bi variable regression were entered to the multivariable logistic regression model. Goodness of fit of the model was assessed by using Hosmer- Lemeshow goodness of fit test. Variables having p-value less than 0.05 in the multivariable model were considered significantly associated with the dependent variable. Odds ratio (OR) with its 95% confidence interval were computed to show the strength of association.

### Results

#### Socio-demographic and reproductive characteristics of study participants

A total of 730 married women were involved with a response rate of 96%. The mean (± standard deviation) age of the married women was 27.8(± 6.2) years. Majorities 712 (97.5%) were orthodox Christians. Almost all 720 (98.6%), were of Amhara tribe. In terms of educational status 23.6% had attended primary formal education and around half (48.5%) of them were illiterates. Majority 516 (70.7%) of the participants were house wives and 299 (41%) of the respondents husbands were farmers. Only 165 (22.6%) had radio or television. Most of the respondents 630 (86.3%) had history of pregnancy and more than half of the respondents 394 (54%) had one or two alive children. More than half 424 (58.1%). of the respondents desire more child. Among those who desire more child, more than half 251 (59.2%) desire one or two children and the reason for desire for majority 347 (81.8%) of the respondents were having few children (Table 1).

**Table 1:** Socio-demographic and reproductive characteristics of married reproductive age women at Janamora district, North Gondar, northwest Ethiopia, 2018

Variable	Category	Number	Percent
Age of married women (n = 730)	18–24	252	34.5
	25–34	350	47.9
	35–44	120	16.4
	45–49	8	1.1
Religion	Orthodox Christian	712	97.5
	Muslim	18	2.5
Educational status	Cannot read and write	354	48.5
	Primary	172	23.6
	Secondary and above	204	27.9
Ethnicity	Amhara	720	98.6
	Others^a^	10	1.4
Monthly income	≤ 22 USD	83	11.4
	23–43USD	344	47.1
	44–54USD	77	10.5
	55–72USD	28	38
	> 72USD	198	27.1
Occupational status of the husband	Government employee	189	25.9
	Daily laborer	45	6.2
	Merchant	125	17.1
	Farmer	299	41.0
	Others^b^	72	9.9
Occupational status of respondent	Government employee	101	13.8
	Daily laborer	12	1.6
	Housewife	516	70.7
	Merchant	36	4.9
	Student	21	2.9
	Others^c^	44	6.0
Have radio or TV	No	565	77.4
	Yes	165	22.6
History of pregnancy	No	100	13.7
	Yes	630	86.3
Number of alive children (n = 630)	1–2	394	54
	3–4	169	23.2
	> 4	18	4.3
Future fertility desire (n = 730)	No	306	41.9
	Yes	424	58.1
Number of desired children	1–2	251	59.2
	3–4	155	36.5
	≥ 4	18	4.3
Reason for future child desire (n = 424)	Have few children	347	81.8
	Need of son	46	10.8
	Need of daughter	29	6.7
	Others^d^	2	0.5
Decision maker on fertility (n = 730)	Wife	98	13.4
	Husband	12	1.6
	Jointly	607	83.2
	Others^e^	13	1.8

^a^Oromo and Tigre, ^b^carpenter, traditional textile workers, nongovernmental employee, ^c^coffee and tea sellers, tella sellers, *ETB* Ethiopian Birr, ^d^Gods prohibition, ^e^God

Currently 12.9% (95% CI 0.1, 0.15) of total respondents utilize LAPMs, among those 91 (96.8%) use implants, followed by female sterilization 02 (2.1) and IUCD 01 (0.1%).

#### Factors affecting the utilization of long acting and permanent contraceptive methods

From multiple logistic regression analysis, the odds of utilizing LAPMs was 4.20 times higher among women who had a good knowledge as compared to those with poor knowledge (AOR = 4.20, 95% CI 1.32–13.39). Furthermore the odds of utilizing LAPMs was 2.51 times higher among women who had government employed husband as compared to merchant (AOR = 2.51, 95% CI 1.1, 5.75). Similarly the odds of utilizing LAPMs was 4.64 times higher among women whose husbands are laborers as compared to merchant (AOR = 4.64, 95% CI 1.67–12.78). Women whose husbands are farmers had 2.34 times increased odds of utilizing LAPMs as compared to merchant. (AOR = 2.34, 95% CI 1.05–5.21). Moreover the odds of utilizing LAPMs was 3.12 times higher among women who were student as compared to housewives (AOR = 3.12, 95% CI 1.05–9.29) (Table 2).

**Table 2:** Multivariable logistic regression of predictors to utilization of LAPMs among married reproductive age women in Janamora District, North Gondar, North West, January 2018

Variables	Utilization of LAPMs		Crude odds ratio (95% CI)	Adjusted odds ratio (95%)	p-value
	Prevalence (%)	Total (N)			
Knowledge of respondent					
Poor knowledge	12.0	676	1	1	
Good knowledge	2.0	40	1.84 (0.82–4.12)	2.06 (0.90–4.69)	0.087
Very good knowledge	35.7	14	4.20 (1.32–12.48)	4.20 (1.32–13.39)	*0.015*
Husband’s occupational status					
Governmental employee	15.3	189	2.65 (1.17–6.00)	2.51 (1.1–5.75)	*0.029*
Daily laborer	22.2	45	4.18 (1.53–11.39)	4.64 (1.67–12.78)	*0.003*
Others	12.5	72	2.09 (0.77–5.68)	2.11 (0.77–5.79)	0.146
Farmer	12.7	299	2.13 (0.96–4.07)	2.34 (1.05–5.21)	*0.038*
Merchant	6.4	125	1	1	
Education of respondent					
Cannot read and write	14.1	354	1	1	
Primary	8.7	172	0.58 (0.32–1.07)	0.59 (0.29–1.19)	0.142
Secondary and above	14.2	204	1.01 (0.61–1.65)	0.70 (0.32–1.52)	0.370
Age of women					
18–24	12.3	252	1	1	
25–34	11.1	350	0.89 (0.54–1.48)	0.80 (0.48–1.38)	0.412
35–44	18.8	128	1.64 (0.92–2.94)	1.41 (0.76–2.59)	0.272
Number of child					
0	12.7	306	0.93 (0.57–1.52)	1.10 (0.65–1.85)	0.731
3–4	10.3	155	0.73 (0.39–1.38)	0.78 (0.39–1.51)	0.455
> 4	27.8	18	2.45 (0.82–7.32)	3.12 (0.97–10.04)	0.056
1–2	13.5	251	1	1	
Occupation of women					
Government employee	19.8	101	1.95 (1.11–3.41)	2.09 (0.99–4.42)	0.540
Daily laborer	25	12	2.63 (0.69–10.00	1.86 (0.45–7.72)	0.390
Others^a^	9	44	0.79 (0.27–2.29)	0.82 (0.28–2.44)	0.720
Merchant	11.1	36	0.99 (0.34–2.89)	1.58 (0.49–5.13	0.443
Student	23.8	21	2.47 (0.87–6.99)	3.12 (1.05–9.29)	*0.041*
Housewife	11.2	516	1	1	

*LAPMs* long acting and permanent methods

^a^ Others includes tea and coffee sellers

### Discussion

This community based cross-sectional study assessed the prevalence of long acting and permanent contraceptive methods (LAPMs) and associated factors among married reproductive age group women’s in Janamora district. Different factors were assessed in this study, and factors like knowledge about LAPMs, husbands occupation and women occupation were found be significantly associated with utilization of LAPMs.

In this study the utilization of LAPMs was found to be 12.9%. This result was in line with other studies conducted in Tigray (12.3%) [17] and Arbaminch (13.1%) [19]. But the it was lower than studies conducted in Debra Markos (19.5%) [20], Sheshamane (28.4%) [21], Nekemt (20%) [10] and Gondar (34.7%) [12]. The low utilization of LAPMs in our study as compared to the others could be due to the low awareness and level of knowledge among the respondents because 92.6% of the participants in this study had a poor knowledge about LAPMs and this may decrease their utilization. Another possible explanation could be the difference in study population and study setting between the studies; because our study was community based and focused only on married reproductive age women but the study done in Shashamene used all reproductive aged women regardless of the marital status. Moreover the study in Gondar was institution based and it only included women who decided not to have more children. Therefore these factors may increase their utilization of LAPMs.

Results of multi-variable analysis showed that women who had good knowledge about LAPMs were 4.2 times more likely to utilize LAPMs as compared to women with poor knowledge. Similar association was found from the studies done in Mekele [17], Jinka [22] and Arbaminch [19]. This could be due to women with a good knowledge might have a better understanding about the special advantages of LAPMs which may increase their utilization.

Women whose husbands occupation is government employee was two and half times likely to utilize LAPMs as compared to women whose husband is merchant. This can be explained that government employees can get information and understand benefits and side effects about LAPMS and influence their wives.

Similarly, a woman whose husbands occupation is daily laborer was 4.64 times increased utilization of LAPMs as compared to a woman whose husband was a merchant. This is supported by study done at Basona Worana district, North Shoa zone [23]. This might be due to their insecure job, low income and unstable life because of this because of this they may prefer long acting contraceptive methods until they get sustainable income and permanent job.

A woman whose occupation is a student was three times more likely to utilize long acting and permanent contraceptive methods as compared to house wife. This finding was supported by study done in Nekemte [10]. This may be due to the fact that students have more access to information which may favor them to use long acting and permanent contraceptives. In addition education may increase their decision making power on reproductive health issues, particularly family planning.

The public health importance of this study is that it identifies the factors which contribute for the increased utilization of LAPMs this give additional information for policy makers, for governmental and nongovernmental stakeholders to improve the utilization of LAMPs and to control the rapid growth of the population at country level.

### Conclusion

Utilization of long acting and permanent contraceptive method is very low in the study area as compared to other studies conducted previously. Among those who used long acting and permanent contraceptive methods, almost all uses implants, a few used IUCD and female sterilization, and few used male sterilization. Based on our findings factors that contribute for better utilization of LAPMs were very good knowledge level of the women, having government employed and daily laborer husband and being a student. The woreda and the Zonal health office should create awareness and give continuous health education to the women about the benefits LAPMs to improve their knowledge about those methods. It is also essential to encourage women for education.

## Limitation of the study

Exclusion of men and unmarred women from this study which may have an effect on the result of the study and failure to establish cause and effect relationship were the limitations of the study.

## Abbreviations

AOR: adjusted odds ratio
CI: confidence interval
EDHS: Ethiopian demographic and health survey
EPI-info: epidemiological information
ETB: Ethiopian Birr
km: kilo meters
IUCD: intrauterine contraceptive device
LAPMs: long acting and permanent methods
OR: odds ratio
SD: standard deviation
SPSS: statistical package for social sciences
TV: television
